# Learning to walk with a wearable robot in 880 simple steps: a pilot study on motor adaptation

**DOI:** 10.1186/s12984-021-00946-9

**Published:** 2021-11-01

**Authors:** Florian L. Haufe, Alessia M. Kober, Peter Wolf, Robert Riener, Michele Xiloyannis

**Affiliations:** 1 Sensory-Motor Systems (SMS) Lab, Institute of Robotics and Intelligent Systems (IRIS), ETH Zurich, Zurich, Switzerland; 2 Spinal Cord Injury Center, Medical Faculty, Balgrist University Hospital, University of Zurich, Zurich, Switzerland

**Keywords:** Exoskeleton, Exosuit, Exomuscle, Motor adaptation, Learning, Familiarization, Robot, Assistance, Walking, Neuromotor control

## Abstract

**Background:**

Wearable robots have been shown to improve the efficiency of walking in diverse scenarios. However, it is unclear how much practice is needed to fully adapt to robotic assistance, and which neuromotor processes underly this adaptation. Familiarization strategies for novice users, robotic optimization techniques (e.g. human-in-the-loop), and meaningful comparative assessments depend on this understanding.

**Methods:**

To better understand the process of motor adaptation to robotic assistance, we analyzed the energy expenditure, gait kinematics, stride times, and muscle activities of eight naïve unimpaired participants across three 20-min sessions of robot-assisted walking. Experimental outcomes were analyzed with linear mixed effect models and statistical parametric mapping techniques.

**Results:**

Most of the participants’ kinematic and muscular adaptation occurred within the first minute of assisted walking. After ten minutes, or 880 steps, the energetic benefits of assistance were realized (an average of 5.1% (SD 2.4%) reduction in energy expenditure compared to unassisted walking). Motor adaptation was likely driven by the formation of an internal model for feedforward motor control as evidenced by the reduction of burst-like muscle activity at the cyclic end of robotic assistance and an increase in arm-swing asymmetry previously associated with increased cognitive load.

**Conclusion:**

Humans appear to adapt to walking assistance from a wearable robot over 880 steps by forming an internal model for feedforward control. The observed adaptation to the wearable robot is well-described by existing three-stage models that start from a cognitive stage, continue with an associative stage, and end in autonomous task execution.

*Trial registration* Not applicable.

**Supplementary Information:**

The online version contains supplementary material available at 10.1186/s12984-021-00946-9.

## Background

Pioneering advances in wearable robotic technology demonstrated that carefully designed assistive devices can improve the efficiency of human walking. Various powered (e.g. [1–5]) and unpowered (e.g. [6–8]) wearable robots have been shown to reduce the energy expenditure during level ground walking, while carrying loads [9, 10], or walking uphill [11, 12]. Other devices, built to address the needs of individuals with a gait disorder, have successfully reduced the mobility impairment associated with stroke [13] or incomplete spinal cord injury [14].

These achievements depend on the users’ ability to integrate the robotic assistance into their movements, or in other words: *learn to walk with a wearable robot*.

Walking is a movement that we practice and refine over years, sometimes at a rate of several thousand steps an hour [15]. An external alteration to this long-learned movement—for example, the physical assistance from a robot—will trigger a process of gradual adaptation as individuals form an internal model of the new movement [16]. There is compelling evidence that locomotor adaptation is accompanied by an improvement in the economy of walking [17–19].

Studies evaluating the economy of walking with a robot typically allotted time for participants to adapt during a familiarization period. Common familiarization durations ranged from 5 [5] to 30 min [10] before the start of the core protocol. These familiarization periods might have been motivated by prior findings showing that energetic benefits nearly doubled after approximately 20 min of training time as participants adapted to the robotic assistance [20]. The range of longer and shorter training durations was likely driven by time and feasibility constraints. All of these training durations were still substantially shorter than previously found adaptation periods of 45 min [21] or even 90 min [19].

In the their study, Sawicki and Ferris [19] showed that an initial 7% increase in energy expenditure within the first minutes of walking with robotic assistance improved to a 10% reduction after three 30 min sessions of walking. This change of 17% is on the same order of magnitude as the energetic benefits of even the latest wearable robots [22], highlighting the importance of user adaptation as major confounder to study results.

So far, studies investigating motor adaptation to walking with a wearable robot were primarily focused on pneumatic [19–21] and electrohydraulic [23] ankle devices. These devices were tethered, lab-based emulators, relying on actuation principles and assistive strategies distinctly different from robots designed for real-world applications [22, 24]. Panizzolo and colleagues [25] recently provided first insights into the energy expenditure during motor adaptation to a more application-focused, untethered exosuit that assisted hip extension. Adaptation was reported to take about 40 min of training time divided into multiple sessions [25], but feasibility constraints confined the authors to investigating metabolic adaptation only. The potentially underlying changes in muscle activation, gait kinematics, and their link to neuromotor control were not captured. Understanding the dynamics of these factors might be crucial to identify biomechanical markers that evidence motor adaptation. These biomechanical markers can help understand the relative contributions of feedback and feedforward strategies to motor control while walking with a robot. They might also help to understand the motor adaptation that is driven by objectives other than the reduction of energy expenditure [26]. While current evidence indicates that minimizing energy expenditure could be the primary objective of motor adaptation in many cases [17, 18], other objectives such as gait stability [26] or preserved kinematics [27] might be prioritized at times.

We seek to better understand how users adapt to assistance from a wearable robot. Such an understanding would be essential to inform effective familiarization paradigms for novice users. Another important benefit of knowing the adaptation time could be that the assistive efficacy of wearable devices is assessed only after users have fully adapted to the assistance. Alternatively, factoring in training time and the resulting degree of adaptation could allow for a more complete discussion of results in comparative reviews of studies involving wearable robots [22]. Optimization approaches in which the robotic controller is adapted in parallel to human motor adaptation, either in a “human-in-the-loop” setup [28] or iteratively offline [29], would also benefit from an improved understanding of motor adaptation to robotic assistance. Finally, an understanding of time needed for adaptation could help to manage user expectations during the initial use of wearable robots. One can speculate that the uptake of wearable robots might have been slowed down by a mismatch between high user expectations and their poor initial performance.

**Fig. 1:**
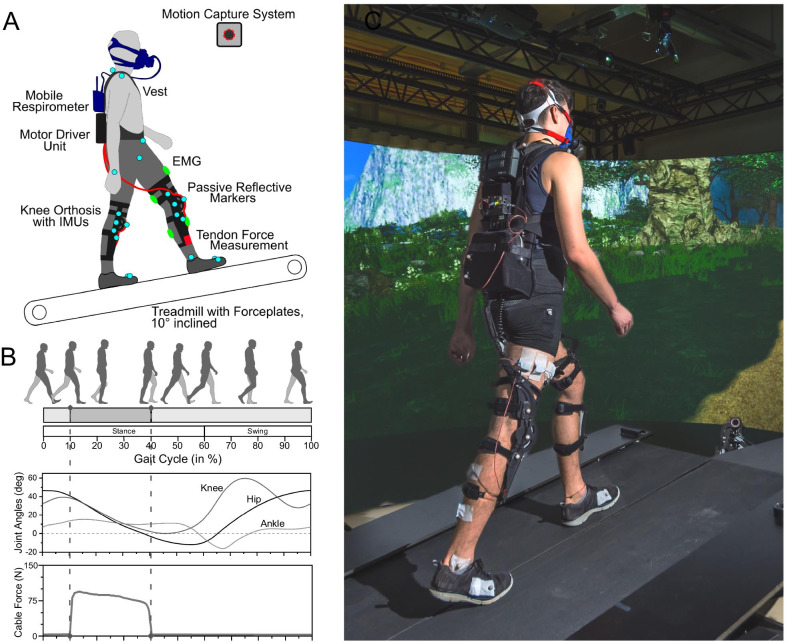
**A** Participants walked on a split-belt treadmill with integrated force plates at a fixed speed of 0.9 m/s and inclination of 10 degrees. A camera system tracked the position of passive reflective markers (light blue). EMG activity (green) was measured on the right leg and a respirometer collected breath-by-breath gas exchange. Tendon forces were measured with a load cell (red) attached to the distal anchor point of the cable on the knee orthosis. **B** The Myosuit assisted hip and knee extension between approx. 10 to 40% of the gait cycle. During the remaining part of the gait cycle, zero forces are applied. **C** Exemplary picture of a study participant in the study setting. Treadmill handrails have been removed for visual clarity but were in place for the experiments

With this study, we aimed to investigate how naïve individuals adapt to walking assistance from a wearable robot, using the Myosuit as model device. The Myosuit assists walking in essential functions [30] by supporting the user’s bodyweight and progression from weight acceptance into late stance (see Fig. 1). On each leg, one cable is routed across the hip and knee joints—exploiting natural extension synergies [31]—and works in parallel with the muscles which have the largest contribution to bodyweight support during walking [32].

Eight male participants (age 27 (22–41) yrs) completed a three-session protocol with 20 min of Myosuit-assisted walking in each session and two 5 min periods of walking with zero forces applied by the Myosuit (zero-force mode) at the beginning and end of each session. They were blinded to the goal of the study. The outcome measures were chosen to connect the processes underlying metabolic adaptation to measures previously associated with neuromotor control. Specifically, we aimed to relate changes in muscular activity and joint kinematics to changes in spatiotemporal stride characteristics [33–35] and arm swing asymmetry (ASA) [36]. The latter two measurements provide an initial estimate of the perceived and actual stability and cortical load while learning to walk with a wearable robot.

## Methods

### Participants

Eight healthy males, age 27 (22–41) yrs, height 180.1 (172.5–186.5) cm, mass 76.2 (72–82.3) kg, mean (range), were recruited as a local convenience sample and participated in the study after they gave their informed consent. We aimed to recruit a homogeneous participant population with respect to gender, height, and mass to reduce the overall experimental variability and circumvent the need to individually adapt the assistance from the Myosuit for each participant. The study design and protocol were approved by the institutional review board of ETH Zurich (EK 2019-N-119). The participants had no previous experience in walking with a wearable robot.

### Wearable prototype

The wearable prototype used in this study (Myosuit Beta, MyoSwiss AG, Switzerland, see Fig. 1) was designed to assist weight-bearing and forward / upward progression during the stance phase of walking.

Assistance to the legs was provided from a backpack-style motor driver unit that housed two electric motors with reduction gears, a battery, and the control electronics. On each leg, a cable was routed from the driver unit posteriorly across the hip joint, laterally across the thighs, and anteriorly crossed the knee joint supported by a cam. The cables were anchored to a 3D-printed polymer knee orthosis attached to the thigh and shank. Cables were made from ultra-high molecular weight polyethylene.

Inertial Measurement Units (IMUs) were placed on both shank and thigh segments and in the motor driver unit to measure linear accelerations and rates of rotation. Based on the IMU sensor data, inter-limb angles and trunk posture were estimated using a five-segment body model. Heelstrike and toe-off events were detected using an algorithm described in [37] and used in conjunction with joint angle estimates to time the cyclic onset and duration of assistive forces.

A textile upper body vest with a waist belt was used to interface the motor driver unit and the knee orthoses to the participants. Two passive elastomer springs that anteriorly crossed the hip joint were only marginally tensioned to counteract downward slipping of the knee orthoses.

### Robotic assistance

Two different control modes were used during the experiments. In assistive mode, a peak cable force of 212 N (measured at the motor winch) was applied between 10 and 40% of the gait cycle, or approximately weight-acceptance and mid-stance, resulting in a distal cable anchor force of 50 to 105 N. During force application, the cable force was further adapted relative to the momentary knee angle (see Fig. 1, and [38] for a more detailed description), where more knee flexion resulted in higher forces. These assistive parameters, constant across all participants of this study, were chosen based on previous human-in-the-loop optimization experiments with a pilot participant representative of this study’s population [39]. In zero-force mode, the cables were driven to avoid slack and minimize interaction forces.

### Experimental protocol

Participants completed a total of three identical sessions separated by at least 48 h, to allow for motor memory consolidation and regeneration [40].The duration between the first and the third visit did not exceed 20 days for any participant in line with previous studies [25]. Each experimental session started with a four-minute period of quiet standing to approximate base metabolism and baseline electromyography (EMG) activity. Afterwards, participants walked for five minutes with zero-forces applied, followed by 20 min in assistive mode and another five minutes in the zero-force mode. We did not warn participants before changing the control mode (assistance/zero-force) of the Myosuit, but only informed them to expect “different levels of assistance over the course of the experiment”. The walking speed was fixed at 0.9 m/s throughout the experiment and the walking surface was inclined at 10°. This setup was chosen since during uphill walking the total positive power of the hip is larger than during level walking [41] and thus the effects of the Myosuit's assistance were expected to be more prominent. The fixed gait velocity was chosen to minimize overall variability and chosen to allow for continuous uphill walking for 30 min without excessive fatigue. During walking trials, participants were asked to only touch the treadmill’s handrails in an emergency. Before sessions, participants were instructed to withhold any food and liquids except for water for at least eight hours prior to the experiment and to refrain from strenuous exercise for at least 24 h prior to the experiment.

### Data collection

Participants walked on a split-belt treadmill (V-Gait Dual Belt, Motekforce Link, The Netherlands) while wearing the Myosuit (see Fig. 1). Ground reaction forces were recorded with two embedded force plates at 1000 Hz and used for stride segmentation. An array of ten cameras (Bonita B10, VICON, UK) was used to track the spatial movement of 28 passive reflective markers placed on the Myosuit and anatomic landmarks at 100 Hz (see Supporting Material for detailed description of marker placement).

The myoelectric activities of m. gluteus maximus (GMAX), m. biceps femoris (BF), m. rectus femoris (RF), m. vastus lateralis (VAS), m. gastrocnemius (GAS), m. soleus (SOL) and m. tibialis anterior (TA) were unilaterally recorded throughout the experiment on the right leg at 2000 Hz. Surface electrodes (Hydrogel/Ag/AgCl, Kendall Arbo H124SG, Covidien, Ireland) were placed in a bipolar configuration with an inter-electrode distance of two centimeters following standard procedures [42]. An additional ground reference signal was measured at the base of the wireless EMG transmitters (Ultium-EMG, Noraxon, USA). Breath-by-breath respiratory data were collected with a portable gas analyzer (K5, COSMED, Italy). The participants’ heart rate was measured with a chest strap (HRM Dual, GARMIN, USA). The linear forces of the right Myosuit cable were measured with a load cell (Miniature S-Beam FSH04416, Futek Advanced Sensor Technology, USA) attached to the distal anchor point of the cable (see Fig. 1) at 100 Hz to allow for the stride-by-stride identification of the Myosuit assistance phase. All measurements were synchronized with analog trigger signals.

### Data analysis

Ground reaction force measurements were filtered (Parks-McClellan, 22 Hz lowpass) and used to identify heelstrike and toe-off events assuming a threshold of 40 N. The gait events were further used to divide other measurement data into individual gait cycles, defined as heelstrike to ipsilateral heelstrike, and stance and swing phase. The time difference between two consecutive ipsilateral heelstrikes was used to calculate stride time over the experimental time of the three sessions. Stride time variability was calculated as the coefficient of variation of the stride time.

Joint angles were calculated based on marker kinematics using biomechanics simulation software (OpenSim 4.0) and an individually scaled version of the Gait 2354 lower extremity model [43] in an inverse kinematics approach. The average joint angle curves for the zero-force mode were obtained by averaging across all steps in both the first and second zero-force conditions, to account for potential order effects.

Raw EMG data were filtered (Parks-McClellan, 20–400 Hz bandpass), the baseline activity was subtracted, and a moving root-mean-square across a time window of 50 ms was calculated. The resulting data were then normalized by the mean of the uppermost 5% of EMG activity for the respective muscle and session following previous literature [44]. The average EMG activation curves for the zero-force mode were obtained by averaging across all steps in both the first and second zero-force conditions.

ASA was calculated as the stride-by-stride ratio of the absolute trajectory lengths of the two reflective markers placed on the left and right wrist following [36], where 0% represents perfect symmetry and positive values left-hand dominant arm swing. For visual clarity, data were then filtered with a combined median (window size n = 3) and Savitzky-Golay filter (polynomial order p = 3, n = 101) over piece-wise continuous experimental segments (first period of zero-force mode (min − 5…0), assistance (min 0–20) and second period of zero-force mode (min 20–25)).

Total energy expenditure was approximated from respiratory data via indirect calorimetry using the formula of Péronnet and Massicotte [45]. We used the respiratory exchange ratio as a proxy for the respiratory quotient (RQ) and verified that participants were in an aerobic exercise regimen (RQ < 1) during the experiments. The resting energy expenditure during quiet standing was subtracted from the total energy expenditure to obtain the physical activity energy expenditure that was reported in this paper. The average total energy expenditure for the zero-force mode was obtained by averaging across both zero-force conditions, to account for potential order effects, such as fatigue.

### Fitting and statistical analysis

Minute-by-minute mean relative change in energy expenditure $\partial EE$ was averaged over all participants and then fitted with a two-term exponential model with four fit parameters $\left\{ {a,b,\tau_{2} ,c} \right\}$ for each session:

$$\partial EE\left( t \right) = \underbrace {{a\exp \left( { - \tfrac{t}{42}} \right)}}_{respiratory\,\,delay} + \underbrace {{b\exp \left( { - \tfrac{t}{{\tau_{2} }}} \right)}}_{motor\,\,adaptation} + \underbrace {c}_{steady - state\,\,EE\,\,reduction}$$

With the first exponential term accounting for the physiological respiratory delay with a time constant $\tau_{1} = 42s$ [46] and the second term capturing the motor adaptation process with $\tau_{2}$ as a fit parameter.

For subsequent statistical analysis, a linear mixed effects model was fitted to the energy expenditure data using least squares regression (Matlab, USA). The model included “session” (possible values: {1,2,3}) and “condition” ({“zero-force”, “assistance”}) as dummy-encoded, categorical fixed effect explanatory variables, “time of assisted walking” (0,1200) as continuous regression variable, and a term “participant” ({P1,…,P8}) as random effect variable. In addition, an interaction between “condition” and “time of assisted walking” was considered.

For the analysis of EMG data, the same linear mixed effects model was fitted to the mean EMG activity in the period from 10 to 40% GC (all muscles), or between 30 and 40% for the detailed analysis of burst activity (RF and VAS). In the EMG models, no interaction terms were considered since an a priori likelihood ratio test indicated that these terms did not increase the variance explained by the model.

ASA and stride time were analyzed with a linear mixed effects model with two dummy-encoded categorical fixed effect variables “session” ({1,2,3}) and “condition”, where the latter encoded minute-by-minute categories throughout the assisted section of the experiment ({MIN1, MIN2, MIN3,…,MIN20}) and additional categories in the first and second period of walking in zero-force mode {ZF1, ZF2}. This modelling approach was chosen to simplistically reflect the anticipated, less continuous changes in ASA and stride time and capitalized on the very accurate measurements of these outcomes.

Joint angle kinematics were compared in a priori defined contrast using a 1-D extension of conventional paired t-tests (statistical parametric mapping, based on random field theory [47]).

## Results

### Metabolic energy expenditure

**Fig. 2:**
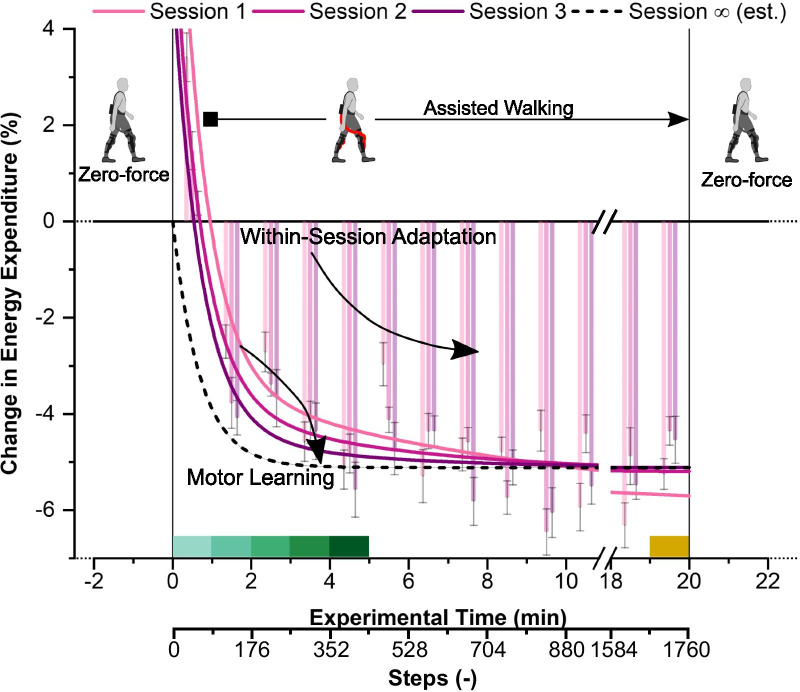
Change in mean (n = 8) energy expenditure during assisted walking compared to walking in zero-force mode. Zero percent corresponds to the average energy expenditure during the two periods of walking with zero forces at the beginning and the end of the respective session. Over the period of assisted walking, energy expenditure is reduced (“Within-Session Adaptation”). This reduction is well described by a two-term exponential fit (solid lines, Session 1,2,3), and occurs faster in later sessions compared to earlier sessions (“Motor Learning”). For visual guidance, an estimate for the change in energy expenditure after extensive training (dashed line) is included. Colored blocks represent the color-coding of individual minutes as used in Fig. 3 and 5

When walking with assistance from the Myosuit the participant’s energy expenditure decreased with time ($F\left( {1,509} \right) = 11,p < 0.01$, see Fig. 2 “Within-Session Adaptation”). This within-session adaptation is well-described with a two-term exponential function—one term for the physiological respiratory delay with $\tau_{1} = 42s$ [46] and one for motor adaptation with $\tau_{2}$ as a fit parameter. The adaptation was faster in later sessions compared to earlier sessions (see Fig. 2 “Motor Learning”). Across sessions, the fitted energy expenditure gradually approached the limit we expect when motor learning is complete and energetic benefits are realized only after the physiological delay.

Myosuit assistance reduced the participants’ energy expenditure compared to walking with the Myosuit in zero-force mode ($F\left( {1,509} \right) = 20,p < 0.001$). The largest average reduction of 6.5% (standard deviation, SD: 3.8%) was observed in minute 10 of assisted walking (Session 2, see Fig. 2). Participants’ individual reductions of energy expenditure ranged from 2 to 12% at this point of the protocol.

### Walking kinematics

**Fig. 3:**
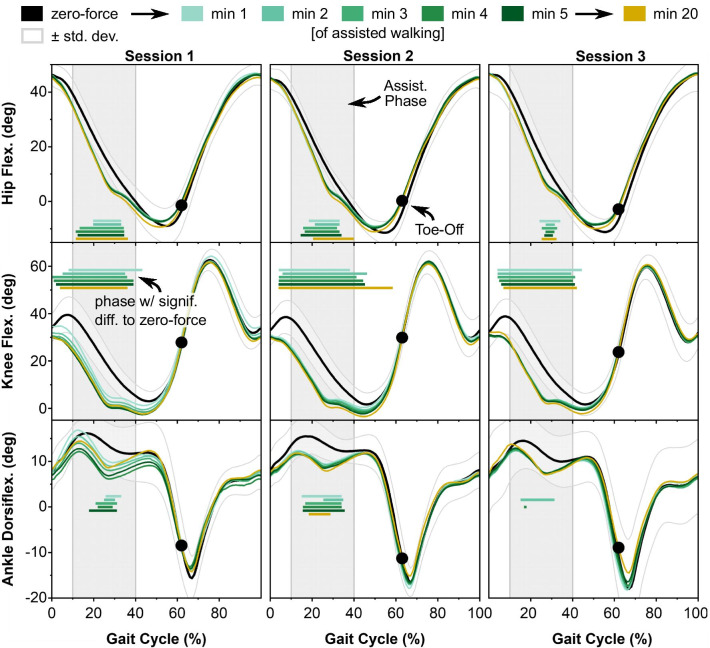
Mean (n = 8) joint angle curves for the hip, knee and ankle joints while walking in zero-force mode (mean in black, +—1 standard deviation, in gray) and minutes 1 to 5 and 20 of assisted walking across Sessions 1 to 3 (colored). Each minute of walking corresponded, on average, to 88 steps. Color-coded horizontal lines mark the period in which a 1-D statistical analysis indicated significant differences between the respective minute of assisted walking and zero-force mode kinematics. The phase between 10 and 40% of the gait cycle in which the Myosuit applies assistive forces is marked in light grey. Most of the kinematic changes occur already within the first minute of assisted walking in Session 1. In later sessions, the convergence to steady state kinematics appears to be faster

Myosuit assistance—applied between 10 and 40% of the gait cycle—led to more extension of the participants’ stance leg. We observed more hip extension, more knee extension, and a trend towards more ankle plantarflexion (see Fig. 3, horizontal lines indicate phases of statistically significant differences compared to zero-force kinematics) when compared to walking with the Myosuit in zero-force mode. The knee was more extended even before the application of Myosuit assistance during the initial weight-acceptance phase (0% to 10% of the gait cycle). Swing phase kinematics were similar when walking with and without assistance from the Myosuit. Toe-off occurred around 62% of the gait cycle throughout all sessions, both in zero-force mode and with assistance from the Myosuit.

These effects of Myosuit assistance on leg kinematics did not change within or across sessions. This was evidenced by the absence of differences between the first minute (Session 1, min 1) and the last minute of assisted walking (Session 3, min 20, all $p = 1$ for hip, knee and ankle). Based on visual inspection, the convergence to the steady-state joint kinematics (min 20) appears to have occurred faster in Sessions 2 and 3 compared to Session 1.

**Fig. 4:**
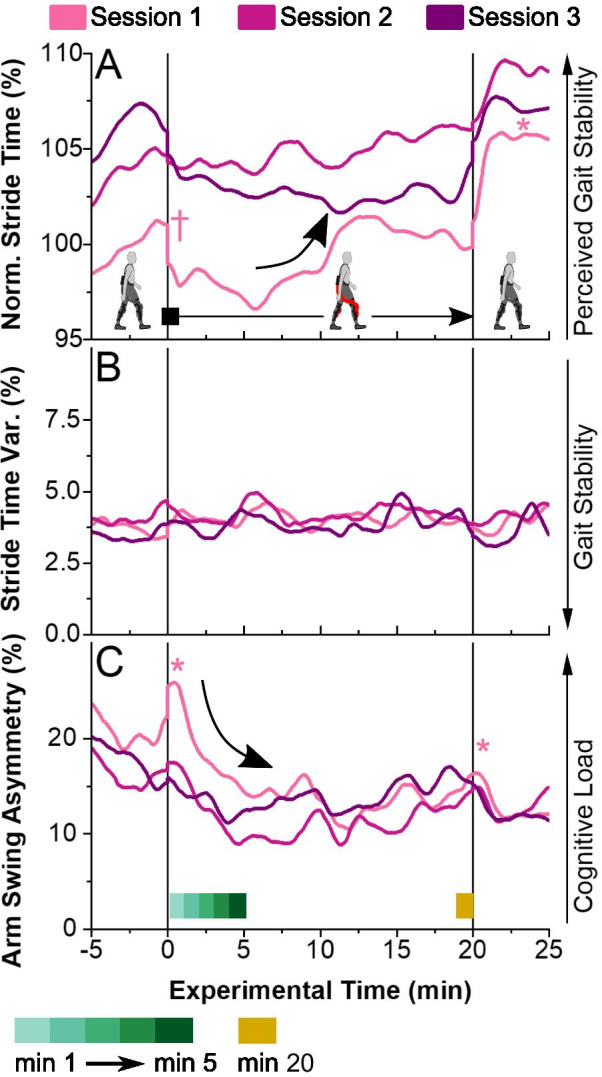
**A** Stride time over the experimental time of the three sessions, normalized relative to the mean stride time of the first zero-force period in Session 1. A longer stride time has been associated with a higher perceived gait stability. **B** Stride time variability represented as the coefficient of variation. Increased stride time variability has been associated with a reduced gait stability. **C** Arm swing asymmetry over the experimental time of the three sessions. An increase in arm swing asymmetry has been associated with increased cognitive load. In both panels, data were calculated as mean over all participants (n = 8). Symbols mark minutes in which outcomes were significantly higher (*) or lower (†) than the experimental mean

Stride time was shorter than the experimental mean in the first minute of assisted walking with the Myosuit ($t\left( {540} \right) = - 3.5,p < 0.001$, see Fig. 4A) and in Session 1 compared to the across-session mean ($t\left( {540} \right) = - 9,p < 0.001$). After approximately 10 min of assisted walking in Session 1, we observed a gradual increase in the mean stride time to a level similar to Sessions 2 and 3. In the second period of walking in zero-force mode, stride time was longer than the experimental mean ($t\left( {540} \right) = 6,p < 0.001$). The stride time variability was constant throughout and across sessions (p > 0.05, Fig. 4B). There was no significant difference in stride time, across sessions, in either one of the zero-force conditions.

Participants reduced the amplitude of their right-arm swing when initially exposed to Myosuit assistance in minute 1 of Session 1 (increase in arm swing asymmetry to 25%; $t\left( {516} \right) = 4.3,p < 0.001$, see Fig. 4C). Another increase in ASA was found in the first 30 s after the assistance was removed ($t\left( {516} \right) = 2.2,p < 0.05$). During all other parts of the experiment, ASA was not different from the experimental mean.

### Muscle activity

**Fig. 5:**
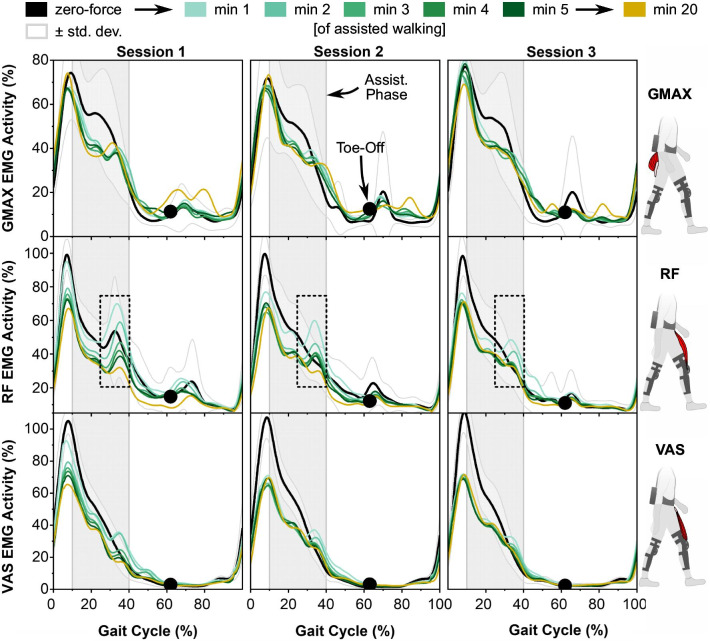
Mean (n = 8) EMG activities over one gait cycle for GMAX, RF and VAS while walking in zero-force mode (mean in black, +—1 standard deviation, in gray) and minutes 1 to 5 and 20 of assisted walking across Sessions 1 to 3 (colored). Each minute of walking corresponded, on average, to 88 steps. The phase between 10 and 40% of the gait cycle in which the Myosuit applies assistive forces is marked in light grey. Dashed boxes mark burst-like RF activity at the end of the Myosuit assistance phase. EMG activities are reduced over the time of assisted walking. The burst-like activity of RF and to a lesser degree VAS is reduced over the time of assisted walking and over sessions

During the part of the stance phase in which the Myosuit provided assistance, we found a reduction of the EMG activities of GMAX ($t\left( {417} \right) = - 10,p < 0.001$), RF ($t\left( {516} \right) = - 12,p < 0.001$), and VAS ($t\left( {489} \right) = - 18,p < 0.001$, see Fig. 5). This general decrease in EMG activity was further associated with a decreasing trend (i.e. more pronounced EMG reduction) over the time of assisted walking for GMAX($t\left( {417} \right) = - 2.2,p < 0.05$), RF ($t\left( {516} \right) = - 8,p < 0.001$) and VAS ($t\left( {489} \right) = - 4,p < 0.001$).

RF, and to a lesser degree VAS, showed burst-like activity towards the end of the Myosuit assistance phase (approx. 30% to 40% of the gait cycle, see dashed boxes in Fig. 5). This activity pattern was reduced over the time of assisted walking (RF: $t\left( {516} \right) = - 10,p < 0.001$, VAS: $t\left( {489} \right) = - 6,p < 0.001$). For RF this was more pronounced in Session 1 than the across-session mean ($t\left( {516} \right) = - 7,p < 0.001$).

GAS and TA showed an increased EMG activity during the Myosuit assistance phase (GAS: $t\left( {561} \right) = 5.3,p < 0.001$, TA: $t\left( {444} \right) = 2.1,p < 0.05$, see Additional files 1 and 2). As for GMAX, RF and VAS, the activity of these muscles was also reduced over the time of assisted walking (GAS: $t\left( {561} \right) = - 8.8,p < 0.001$, TA: $t\left( {444} \right) = - 3.3,p < 0.001$). The EMG activities of BF and SOL were not different during assisted walking compared to walking in zero-force mode (BF: $t\left( {455} \right) = - 1.3,p = 0.18$, SOL: $t\left( {561} \right) = 0.74,p = 0.46$).

## Discussion

### Motor adaptation plateaus after 880 steps (10 min of assisted walking)

The energetic benefits of Myosuit assistance appear to be fully realized within the first ten minutes of continuous walking with assistance, or after around 880 steps. We interpret the gradual decrease in the participants’ energy expenditure up until this point as evidence of ongoing within-session motor adaptation (see Fig. 2). After ten minutes of assisted walking (Session 1), the energy expenditure was at the same level as in all following sessions after 10 min or 20 min of assisted walking (see Fig. 2).

### Adaptation period is consistent with previous work

Table 1 lists studies that specifically report metabolic and/or muscular effects of learning to walk with a wearable robot, with emphasis on walking conditions, assisted joint, mode of assistance, metabolic effect size and adaptation time. A rigorous comparison of our work with existing literature is not yet possible because of differences in experimental procedures (walking conditions, walking speeds) and characteristics of the wearable robots (assisted joints, controller, and peak support) used to conduct the investigations. A further obstacle is given by most studies using time as a unit measure of adaptation: developmental studies suggest that the number of steps, rather than time, are a more appropriate explanatory variable for walking ability in infants [15]; further studies would be needed to investigate if this still applies to adults when learning to walk with a wearable robot. Despite these differences, a qualitative meta-analysis of existing results would help to put our results into context.

**Table 1:** Studies that have investigated human adaptation processes while walking with assistance from a wearable robot

Study	Walking speed (m/s)	Supported DoF	Controller	Peak support	Metabolic effect size	Adaptation time (min)
Sawicki [19]	1.25	Ankle PF	Myoelectric	100 W	− 10%	~ 90
Galle [20]	1.36	Ankle PF	Kinematic	245 W	− 16.6%	18.5
Koller [48]	1.2	Ankle PF	Myoelectric	144 W	− 17.8%	~ 30
Panizzolo [25]	1.5^b^	Hip E	Kinematic	300 N	− 10.5%	~ 10
Here	0.9^a^	Hip E, Knee E	Kinematic	100 N	− 5.1%	10 (~ 880 steps)

*PF *Plantarflexion, *E* Extension

^a^Inclined 10°; ^b^Loaded walking (20.4 kg)

The metabolic adaptation time of 10 min or 880 steps, found here, is comparable to that found in the work of Galle et al*.,* in which full assistive benefits were found after 18.5 min (ankle exoskeleton [20]) and to the results in Panizzolo and colleagues, where learning to walk with a soft hip exosuit required approximately 10 min [25], albeit at a higher walking speed. The authors of the second study concluded that adaptation was only complete after 40 min of walking. However, their results showed no across-session difference for minimum energy expenditure (p = 0.15), raising the question if adaptation was not in fact already complete after the first 10 min of walking.

Another study [19] that reported a much longer adaptation period of 90 min also observed a larger energetic adaptation magnitude, of 10%. Interestingly, this study also found a reduction of energy expenditure across sessions for walking in the zero-force condition, in contrast to [25] and our present study, where energy expenditure in the zero-force condition was constant across sessions. We interpret a change in energy expenditure without robotic assistance as sign of a concurrent adaptation that was unrelated to the assistance of the wearable robot, e.g. adaptation to treadmill walking or walking with body-worn measurement systems. Thus, the very long adaptation period of 90 min might include these concurrent adaptation processes, and overestimate the time required to adapt to walking with a wearable robot. Given that there are no between-session changes in the energy expenditure in zero-force mode we conclude that our study is not affected by the sensorimotor familiarization to treadmill walking to the same degree.

Finally, replicating the protocol in [19] but including an adaptation feature in the controller of the robotic device, Koller et al. [48] found adaptation times one third smaller than those in [19], while reaching an average metabolic effect size of − 17.8%. This result seems to suggest that control strategies (especially kinematic versus myoelectric controllers) play a fundamental role in determining the timescale of the human learning process.

### Participants form a feedforward-model of walking with robotic assistance

In an extension of previous work, we measured the participants’ gait kinematics (see Fig. 3) and muscle activities (see Fig. 5) during the motor adaptation process. Kinematics show the most pronounced adaptation within the first minute, around 88 steps, of assisted walking. This fast adaptation is in line with previous work where kinematic and muscular adaptation was found even on a step-by-step level after mechanical forces were applied [49]. After the first minute of assisted walking, kinematic adaptation was largely complete and gait kinematics were consistent throughout the experiment (see Fig. 3). The rate of adaptation with respect to muscle activation was also most pronounced in the first minutes of assisted walking, but a further reduction in activity was observed for GMAX, RF and VAS afterwards until approximately minute 5 (440 steps) of assisted walking (see Fig. 5).

Concurrent with the high initial rate of muscular and kinematic adaptation, we observed a sharp increase in ASA (see Fig. 4C). An increase in ASA has been linked to an increased cortical load under motor-cognitive dual tasking conditions [36]. Previous work further detailed that the initial motor adaptation to walking with a robot is driven by sensory feedback that allows users to form an internal task model for feedforward motor control [16, 49]. The increased ASA observed over the first minute of assisted walking in this study hence suggests a high level of cortical involvement in feedback-driven motor control and in the simultaneous formation of a feedforward task model [16]. Consistent with this notion, a similar albeit smaller increase in ASA was found in the period immediately after assistance was removed, akin to a wash-out period, in which participants reverted to regular walking in zero-force mode.

Further evidence for the formation of a feedforward model for motor control is found in the reduction of burst-like muscle activity at the end of the Myosuit assistance phase. Initially, RF and VAS showed an increase in activity towards the end of Myosuit-assistance phase (see Fig. 5, dashed boxes). This increase temporally coincides with the cyclic end of the assistance phase of the Myosuit. During this phase, GMAX and VAS are the main biological contributors to bodyweight support and forward progression in walking, and RF acts in parallel to these muscles to extend the hip (sic) and knee [50]. Burst-like activity of VAS and RF hence suggests that these muscles were recruited to provide additional weight-bearing support and stabilization in the sagittal plane in response to the (initially unexpected) end of Myosuit assistance. We follow that as participants internalized the timing of assistive forces into their model of walking with Myosuit assistance, this feedback-driven burst in muscle activity was gradually reduced through appropriate feedforward control contributions (see Fig. 5). In addition to portions driven by an internal model, feedforward control contributions might also comprise a portion that aims to continuously reduce muscle activation, or “slack” the human motor system [51].

Upon removal of assistance, however, muscular activation patterns quickly (2–3 steps) went back to baseline characteristics, suggesting that participants returned to their primary internal model of musculoskeletal mechanics. These results corroborate the findings from Gordon and Ferris [21], where a similar effect was observed for the soleus muscle, in participants walking with an ankle exoskeleton. Unlike the results reported in Farrens et al. [52] or Emken and Reinkensmeyer [16], however, we did not observe lasting post-adaptation training effects.

### Perceived gait stability might partially recover with adaptation

A shift towards shorter stride times was observed in the first minute of assisted walking (see Fig. 4A), which—on a treadmill running at fixed speed—equals shorter stride lengths. Based on previous literature [33, 35], we interpret these changes in stride length as potential changes in the perceived gait stability of participants. Myosuit assistance appeared to initially decrease perceived gait stability, an effect that was partially reversed as participants formed a model of walking with assistance over the first 10 min of assisted walking (see Fig. 4). In the final period with zero forces, stride time increased again, further supporting a causal relation of stride time changes to Myosuit assistance. The absence of changes in the stride time and hence length variability (see Fig. 4B), a metric previously associated with the (objective) gait stability [33], indicates that Myosuit assistance did not affect the participants’ gait stability.

Mechanisms other than perceived stability might confound changes in stride length. Humans have been shown to adapt stride length to reduce their energy expenditure during walking in the presence of external forces [18]. Hence, it might be that walking with Myosuit assistance energetically favors shorter stride lengths compared to walking in zero-force mode. Yet, this mechanism would fail to explain why participants consistently reduced their stride length in the first minute of assisted walking, and only slowly reverted to a steady state across the minutes thereafter. Stride length adaptation, as a (potentially exploratory) response to a shift in the energetic optimum, would be expected to occur on a much shorter time scale, i.e. over tens of seconds [18].

Alternatively, shorter stride length has also been associated with increased cognitive load in dual tasking scenarios [53]. This alternate interpretation would be consistent with the observed increase in ASA and additionally support an increase in cognitive load in the first minute (88 steps) of assisted walking.

### Adapted motor skills partially retained between sessions

Evidence for an increased cognitive load and reduced perceived gait stability was observed at the beginning of Session 1, but not in subsequent sessions (see Fig. 4). In addition, the convergence of joint kinematics and muscle activities towards a within-session steady state appeared slower in Session 1 than in subsequent sessions (see Figs. 3 and 5). We interpret these effects as motor learning; that is, as a sign that participants partially retained their task model and adapted motor skills from Session 1 across subsequent sessions.

This partial retention that required a period of re-adaptation at the beginning of later sessions is consistent with the findings of Panizzolo et al. [25]. Other work reported a much higher skill retention that allowed for near-continuous learning across sessions [19]. As mentioned before, though, this study’s results might have been confounded by adaptation to factors other than robotic assistance, given that the energy expenditure in the zero-force mode also decreased over time. The intervals between sessions in [19, 25]—3 to 5 days—were comparable to the ones in our study and hence do not explain the difference in skill retention. In light of this, we conclude that an energetic minimum might generally only be reached after re-adaptation, even if the respective participant had practiced the same task in previous training sessions before. It remains an open question for research if this need for re-adaptation would eventually be diminished after extensive practice and associated motor memory consolidation.

### Motor adaptation matches a three-stage model

While our study was not designed to validate any specific motor adaptation model, our findings are consistent with models from literature (e.g. [54]) that suggested to distinguish three stages of skill acquisition. These models describe the motor learning process from a largely cognitively driven stage over an associative stage to autonomous task execution:

#### Cognitive stage

Major kinematic and muscular adaptation at high rates. Accompanied by increase in cortical load, likely due to initial formation of an internal model of the task.

Duration approx. 100 steps or just over 1 min.

#### Associative stage

Continuing formation of an internal model for feedforward movement control. Reduction of feedback-driven muscle activation at end of assistance. Full energetic benefits of robotic assistance are realized at the end of this period.

Duration approx. 880 steps or 10 min.

#### Autonomous stage

Only minor adaptation of joint kinematics and muscle activities. Participants now at ease with the robotic assistance, perceived gait stability increased compared to the previous stages. No further changes in energy expenditure on the investigated time scale.

We propose to routinely include outcome metrics such as ASA or changes in stride length in future studies of wearable robots to contribute towards an estimate of skill acquisition progress across these stages. Even outside of laboratories, changes in ASA and stride length could be approximated though inertial measurement units placed on the participants’ wrists and shanks. Such information would add important context to study findings and increase the comparability of results.

It remains an open question how this three-stage model transfers to user populations with a neuromotor impairment, a key target group for lower-limb wearable robots. Previous findings [55] revealed that the ability of stroke survivors to form models for feedforward motor control is impaired. Hence, one might speculate that some individuals with a neuromotor impairment might not be able to progress from the associative stage to fully autonomous task execution, but this remains to be verified in future work.

### Study limitations and recommendations

It appears that mean EMG activity and gait kinematics considered separately do not explain the entire variation in energy expenditure. The continuing reduction of burst-like activity of GMAX, RF and VAS after the first ten minutes of walking did not translate to any (measurable) changes in energy expenditure. This is in parts expected, e.g. due to the fact that muscle efficiency depends on muscle length changes during activity [56]. Moreover, one can assume that EMG measurements can resolve small changes in local muscle activity that do not result in changes in the global energy expenditure measured via respirometry. To this end, dynamic simulations that combine the analysis of EMG activity and gait kinematics could allow for a more integral analysis of kinematic, muscular, and energetic adaptation.

Further, our current study did not include “catch-trials”, i.e., unexpected steps without assistance in the 20-min period of assisted walking. An observation of the leg kinematics and muscle activities during such catch-trials might provide further evidence for the formation of an internal model for feedforward motor control [57]. Transfer tasks (e.g., changing the walking speed, pitch, or surface properties) could further illuminate the robustness of such an internal model. Finally, dual-tasking scenarios (e.g. a Stroop task [36] during walking) comprised within future learning studies could strengthen the evidence for an association of cognitive load and increased ASA.

Finally, this study focused on a homogeneous sample and controlled conditions: we recruited healthy males in a narrow range of mass and height; the Myosuit specifically supported hip and knee extension with peaks forces of 212 N during mid-stance; participants walked uphill at a fixed speed. Most of these choices were driven by practical considerations and are a first necessary step before investigating motor learning processes on a more heterogeneous sample and less controlled conditions; they do, however, limit the generalizability of our findings.

## Conclusions

Our results highlight the importance of considering motor adaptation during scientific investigation of wearable robots. In comparative analyses of different devices (e.g. [22]), adaptation time and repetition count (e.g. steps) will likely be a major performance confounder and should be clearly reported, ideally with respect to a common skill acquisition model. Optimization approaches such as human-in-the-loop techniques need to allow for sufficiently long intervals between control changes to estimate the energy expenditure not only after accounting for the respiratory delay, but also after energetic adaptation is (mostly) complete. Our findings inform the ongoing trade-off between achieving rapid energy expenditure estimation and allowing for a meaningful degree of adaptation to the tested control condition.

This warrants for caution when evaluating the effect of similar powered orthoses on impaired individuals: one should allow for adaptation times at least in order of the ones found here before drawing conclusions on the efficacy of the device. We believe that such knowledge could help to manage the expectations of medical professionals and patients, thus preventing premature abandonment of technology that could ultimately benefit the user. Future studies on people with mobility disorders will help to clarify these hypotheses.

We demonstrated that relatively easily accessible biomechanical markers such as ASA and stride length might help to inform investigators about the progress of skill acquisition and motor adaptation. In our study, these markers, in conjunction with muscle activity measurements, suggest that the initial formation of a task model for feedforward motor control took approx. 10 min or 880 steps of assisted walking in young, unimpaired participants. A period of this length should be included in future study protocols that investigate similar modes of assistance, before energetic assessments are made and repeated on every subsequent testing day given the observed partial skill retention. Humans might adapt faster to smaller external alterations such as gradual changes in force magnitude or timing during optimization experiments. Adaptation periods might differ for wearable robots that more prominently assist the propulsion phase of walking instead of the weight-bearing phase.

The fast and marked departure from the kinematics of walking in zero-force mode suggested that preserving kinematics was not an objective of motor adaptation. Instead, our findings were consistent with energy expenditure being a key objective of adaptation, perhaps with gait stability as a secondary one.

Future research needs to clarify how to improve skill retention between sessions, and what minimum training frequency is needed to retain learned skills. Another open research question is how training intensity affects motor adaptation, e.g., if walking at a higher cadence would result in faster adaptation, timewise or even with a lower number of steps.

Finally, motor adaptation might also occur on a much longer time scale, over years of continuous practice. To fully understand the lasting relevance and long-term progression of motor adaptation to wearable robots, a considerably longer observation period than realized in this study or previous studies would be of interest. One can speculate that with the advent of more habitual at-home use of wearable robots, such studies will eventually become more feasible. Until then, it remains unclear if anyone has yet learned to fully utilize the potential of wearable robots—in this instance, indeed only time, or more steps, will tell.

## Supplementary Information

**Additional file 1.** Details on optical marker placement, the muscular activity of the gastrocnemius and tibialis anterior, and missing data.

**Additional file 2.** Learning to walk with a wearable robot in 880 simple steps: a video summary.

## Data Availability

All the data relevant to this study are presented within the manuscript and the additional files.

## Abbreviations

SD: Standard deviation
ASA: Arm swing asymmetry
IMU: Inertial measurement unit
EMG: Electromyography
GMAX: M. gluteus maximus
BF: M. biceps femoris
RF: M. rectus femoris
VAS: M. vastus lateralis
GAS: M. gastrocnemius
SOL: M. soleus
TA: M. tibialis anterior
