# Supplementary material for: Learning to walk with a wearable robot in 880 simple steps: a pilot study on motor adaptation
Source: J Neuroeng Rehabil. 2021 Nov 1;18:157. doi: 10.1186/s12984-021-00946-9 (PMC8561899; doi:10.1186/s12984-021-00946-9)
Supplement: Supplementary file 1 — Additional file 1. Details on optical marker placement, the muscular activity of the gastrocnemius and tibialis anterior, and missing data. [file 12984_2021_946_MOESM1_ESM.pdf]

Additional file

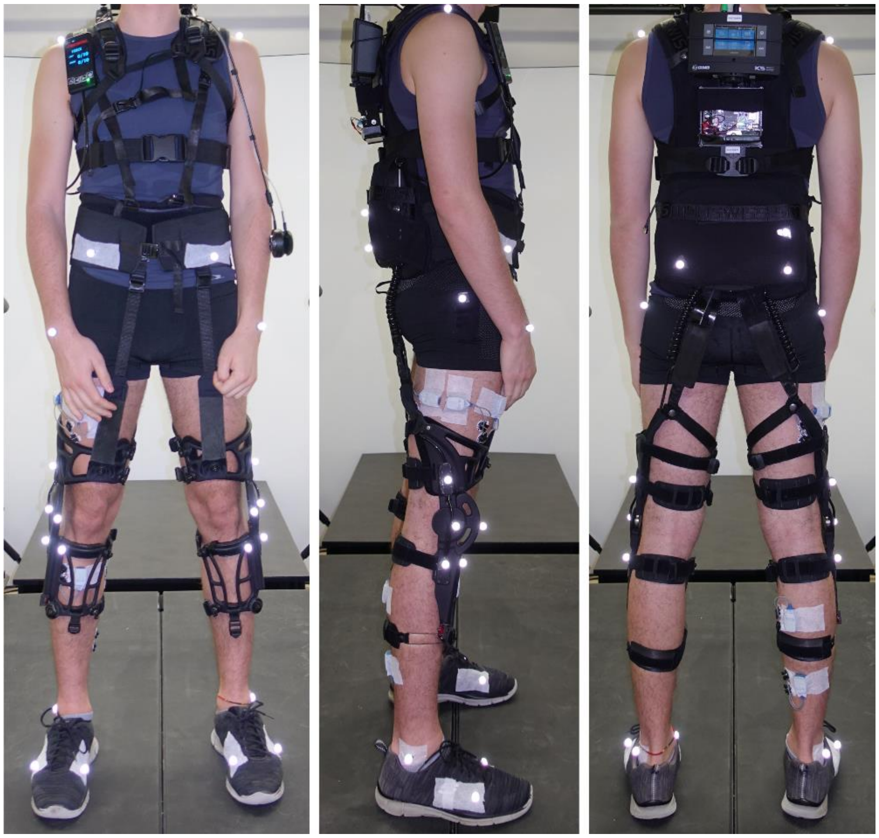

Figure A. Frontal plane and sagittal plane pictures of an exemplary participant to illustrate the placement of reflective markers during experiments.

Reflective Marker Placement

A total of 28 passive reflective markers were attached to the lower extremities, the trunk, the wrists and parts of the Myosuit.

Markers were bilaterally placed on the first and fifth metatarsals, the lateral malleoli, the greater trochanter head, the anterior superior iliac spine and the acromia. On each knee brace, five markers were placed in suitable locations to approximate thigh and shank dynamics. Additional markers were placed on the spinous process of the seventh cervical vertebra and bilaterally between the processus styloideus ulnaris and the radius. Three markers were placed on the backworn tendon driver unit to track the displacement upon force application but disregarded during the reconstruction of joint angle kinematics due to pronounced relative movement between the unit and the trunk.

Muscle activities of GAS and TA

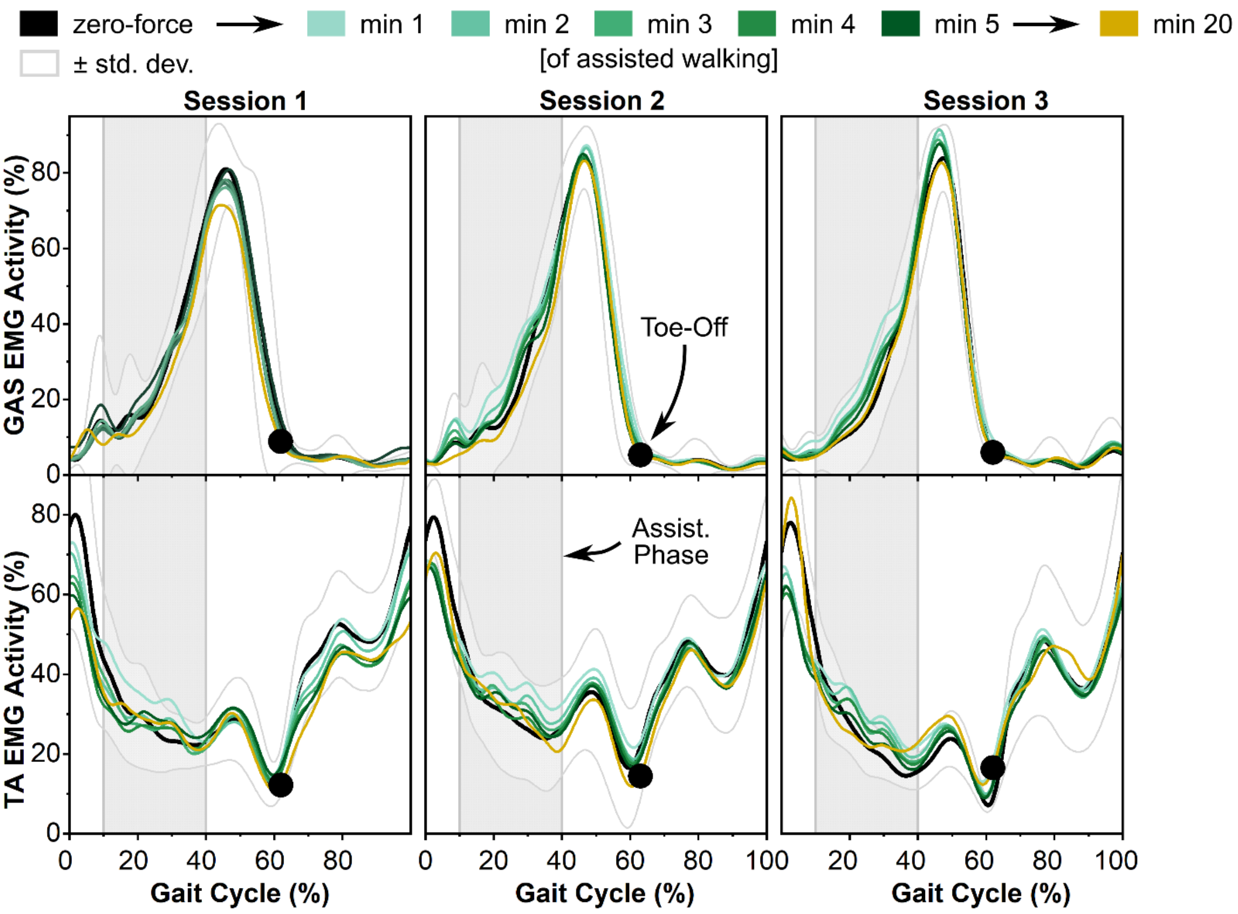

Figure B. Mean (n=8) EMG activities over one gait cycle for GAS and TA while walking in zero-force mode and minutes 1 to 5 and 20 of assisted walking across Sessions 1 to 3. The phase between 10 % and 40 % of the gait cycle in which the Myosuit applies assistive forces is marked in light grey.

Missing Data

When experimental errors occurred that required the participants to stop walking, we prioritized to control for total exposure time and disregarded all data during a transient restart period of two (if stop was in the second half of a minute, three) minutes. We did not repeat any portions of the sessions. The missing data for some outcomes is reflected in the varying degrees of freedom reported in our statistical tests.

During session 1, participant LWP_03 stepped into the treadmill light barrier which triggered a full emergency stop. We disregarded minutes 9 and 10 following the full stop event for all outcome metrics for this participant. During session 2 of participant LWP_06, we briefly stopped the tread-mill to reattach passive reflective markers that fell off and disregarded all data from the subsequent

two minutes (14 and 15). During session 1 of participant LWP_08, we stopped the treadmill to adjust fittings of the Myosuit that had come undone. We disregarded minutes 10, 11 and 12 following this intermittent pause. During session 3 of participant LWP_08, we briefly stopped the treadmill to retrieve an EMG electrode that fell off and irritated the participant. We disregarded all data from the subsequent three minutes (11, 12, 13).

The energy expenditure data from session 2 of participant LWP_09 was disregarded because of a pronounced drift over time that we did not observe for any other session or participant and attributed to a malfunctioning of the respirometer. The arm swing asymmetry data from LWP_04, session 2, was disregarded as the participant reported an itching sensation that caused him to regularly scratch himself with his right hand.

The following EMG data were corrupted and disregarded, typically because of electrode detachment, cyclic force application on the electrode due to a shift of the Myosuit, or battery issues:

**Muscle**	**Participant**	**Session**
RF	LWP_04	2
RF	LWP_08	1
VAS	LWP_04	1
VAS	LWP_04	2
VAS	LWP_05	1
GMAX	LWP_03	3
GMAX	LWP_04	3
GMAX	LWP_07	2
GMAX	LWP_08	1
GMAX	LWP_08	2
GMAX	LWP_08	3
BF	LWP_05	2
BF	LWP_06	1
BF	LWP_07	3
TA	LWP_03	2
TA	LWP_03	3
TA	LWP_06	2

In addition, the GMAX electrodes of LWP_05, LWP_06 and LWP_07 detached within the last three minutes of their respective session 3. In order to efficiently utilize the available data and avoid

exclusion of these participants from GMAX EMG outcomes, we decided to show the EMG activity for GMAX of minute 17 in session 3 instead of minute 20 in Figure 5.

Supplementary Video

See separate additional file.
